# Gene Knockout and Overexpression Analysis Revealed the Role of N-Acetylmuramidase in Autolysis of *Lactobacillus delbrueckii subsp. bulgaricus* Ljj-6

**DOI:** 10.1371/journal.pone.0104829

**Published:** 2014-08-11

**Authors:** Xiao-Yang Pang, Wen-Ming Cui, Lu Liu, Shu-Wen Zhang, Jia-Ping Lv

**Affiliations:** Key Laboratory of Agro-Food Processing and Quality Control, Institute of Agro-Food Science and Technology, Chinese Academy of Agricultural Science, Beijing, P.R. China; Ludwig-Maximilians-University Munich, Germany

## Abstract

Autolysis of lactic acid bacteria (LAB) plays a vital role in dairy processing. During cheese making, autolysis of LAB affects cheese flavor development through release of intracellular enzymes and restricts the proliferation of cells in yogurt fermentation and probiotics production. In order to explore the mechanism of autolysis, the gene for the autolytic enzymes of *L. bulgaricus*, N-acetylmuramidase (*mur*), was cloned and sequenced (GenBank accession number: KF157911). *Mur* gene overexpression and gene knockout vectors were constructed based on pMG76e and pUC19 vectors. Recombinant plasmids were transformed into *L. bulgaricus* ljj-6 by electroporation, then three engineered strains with pMG76e-mur vector and fifteen engineered strains with pUC19-mur::EryBII were screened. The autolysis of the *mur* knockout strain was significantly lower and autolysis of the mur overexpressed strain was significantly higher compared with that of the wild type strain ljj-6. This result suggested that the *mur* gene played an important role in autolysis of *L. bulgaricus*. On the other hand, autolytic activity in a low degree was still observed in the *mur* knockout strain, which implied that other enzymes but autolysin encoded by *mur* were also involved in autolysis of *L. bulgaricus*.

## Introduction

Lactic acid bacteria are used as starter cultures for dairy fermentation and therefore their lysis is of special interest. In cheese making, ripening is an extremely important process since it defines the flavor and texture of the cheese, which differentiates the many varieties. The process is very slow, generally between three weeks and two (or more) years. This makes cheese making an expensive business. Several technological methods have been used to accelerate ripening, such as increasing the ripening temperature, the use of modified starter cultures, and the addition of exogenous enzymes [1], [2]. Lysis of the starter strains during ripening results in the release of cytoplasmic peptidases, lipases, and other enzymes involved in amino acid catabolism, into the cheese curd [3], [4]. It is thought that these enzymes accelerate peptidolysis and remove bitter-tasting peptides. Thus, increasing starter LAB autolysis is considered essential to accelerate cheese ripening [5]. In yogurt making, autolysis of lactic acid bacteria can lead to low number of live strains in starter cultures, such that reducing starter LAB autolysis can result in more efficient yogurt starter cultures. Therefore, understanding the mechanism of LAB autolysis has important significance in dairy industry.

**Figure 1:**
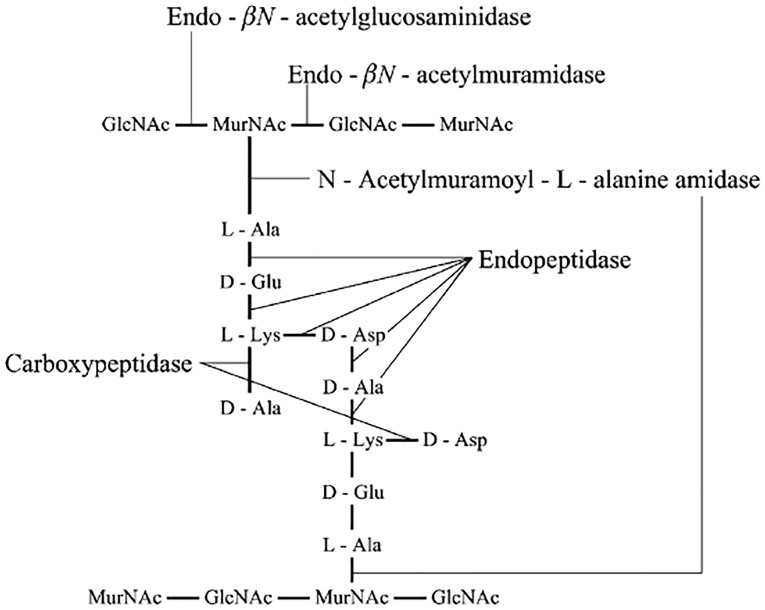
A general representation of the lactococcal peptidoglycan structure showing the specificities of the different peptidoglycan hydrolases [2].

Generally, autolysis is mainly caused by lactic acid bacteria autolysins which are defined as endogenous enzymes that hydrolyze covalent bonds in the peptidoglycan of the cell wall. Five types of enzymes with lytic activity against peptidoglycan have been described for Gram-positive bacteria [6]. Figure 1 is a diagram of the peptidoglycan structure found in L. lactis with the five different types of peptidoglycan degrading activity indicated [2]. Mercier [7] cloned and sequenced the gene for N-acetylmuramidase and showed by zymogram analysis that it was the only autolysin expressed in the plasmid-free strain L. lactis subsp. cremoris MG1363. The N-acetylmuramidase open reading frame in MG1363 encodes a protein with a predicted molecular mass of 46,564 Da, which after cleavage of an N-terminal leader sequence would result in a mature N-acetylmuramidase with a predicted molecular mass of 40,264 Da.

The aim of the present study was to clone and sequence the N-acetylmuramidase gene from the *L. bulgaricus* Ljj-6, exploring the relationship between N-acetylmuramidase gene and *L. bulgaricus* autolysis, and laying the foundation for the directional control of lactic acid bacteria autolysis in future studies.

## Materials and Methods

### Bacterial cultures, media and growth conditions

The parental strains and plasmids that were used in this study are listed in Table 1. Escherichia coli cells were grown in Luria-Bertani broth (LB) medium with aeration at 37°C. L. *bulgaricus* were routinely grown at 37°C in Man-Rogosa-Sharpe (MRS) medium (Beijing Land Bridge Technology Co., Ltd. CM187) under static conditions. MRS was autoclaved for 15 min at 121°C. The strain morphology was observed by light microscopic Olympus CX41.

**Table 1:** Bacterial strains and plasmids used in this study.

Strain or plasmid	Relevant genotype or description	Reference and/or source
Strains		
*E. coli* DH5α	F^−^, φ80d *lac*Z ^△^M15, ^△^(*lacZYA*-*argF*) U169, *deoR*, *recA*1, *endA*1, *hsdR*17 (rk^−^, mk^+^ ), *phoA*, *supE*44, λ^−^, *thi*-1, *gyrA*96, *relA*1	TaKaRa
*L. bulgaricus ljj-6*	Wild-type *L. bulgaricus* isolated from Yogurt samples; low rate of autolysis	this study
*L. bulgaricus* 11842	Wild-type *L. bulgaricus* with normalrate of autolysis	ATCC 11842
*L. bulgaricus k12*	*mur* knockout mutant of *L. bulgaricus*ljj-6; *mur::EryBII*	this study
*L. bulgaricus O3*	*mur* overexpression mutant of *L. bulgaricus* ljj-6; ljj-6 introductionof a functional *mur* gene on expression vector pMG76e;	this study
Plasmids		
pMD18T	clone vector; Amp^r^	TaKaRa
pMG76e	pMG36e derived integration vector; Em^r^	College of food science and Nutritional Engineering, China Agricultural University
pMD18T-mur	pMD18T derived integration vector containing the *mur*ljj-6 gene with *sal*I, *sph*I restriction enzyme sites; Amp^r^	this study
pMG76e-mur	pMG76e derived expression vector containing the *mur*ljj-6 gene; Em^r^	this study
pUC19	clone vector; Amp^r^	TaKaRa
pUC19-mur	pUC18 derived integration vector containing the *mur*ljj-6 gene with *BstEII*, *HinCII* restriction enzyme sites; Amp^r^	this study
pUC19-mur::EryBII	pUC18-mur; Em^r^; Amp^r^	this study

### DNA manipulations

Routine molecular biology techniques were performed according to standard procedures. Restriction and modifying enzymes (New England Biolabs) were used as recommended by the manufacturer. Plasmid DNA was prepared from *E. coli* and *L. bulgaricus* cells by use of BIOMIGA Plasmid Miniprep kits (PD1211-01). Chromosomal DNA was isolated from *L. bulgaricus* cells by use of BIOMIGA Bacteriall gDNA kits (GD2411-01).

### Amplification of the *mur* gene

DNA was extracted from *L. bulgaricus* ljj-6 using the sodium dodecyl sulfate/proteinase K/cetyltrimethylammonium bromide (CTAB) method [8] with minor modifications. The *mur* gene was amplified from the DNA template by PCR with ExTaq DNA polymerase (Takara) and primers containing an *sal*I site and an *sph*I site (Primer 3,4 in Table 2). The PCR reaction mixtures contained 250 ng of genomic DNA as template, 100 pmol of each primer, 5 U of ExTaq DNA polymerase, 12.5 mM MgCl_2_, and 200 mM each dNTP in deionized water to a final volume of 50 mL. The reaction was performed using the PCR thermocycler (Hangzhou Bioer Technology Company, China) with a program consisting of 1 cycle at 95°C for 4 min, then 35 cycles of 95°C for 1 min, 55°C for 1 min, and 72°C for 2 min, and finally 1 cycle of 72°C for 8 min. Products were quantified by comparing the intensity of the product DNA band to that of a 2000 bp marker standard in 1% agarose. Each DNA fragment was ligated into the pGEM-T Easy vector (Promega) according to the manufacturer’s instructions at 16°C overnight. The recombinant plasmid was then transformed into *Escherichia coli* DH5α competent cells. Positive clones of transformed cells were selected and sequenced at Shanghai Invitrogen Company, China.

**Table 2:** Primers used in this study.

No.	Primer	Sequence	Reference
1	Lb-murF1	5′-ATGGCTGGACACAGAAA-3′	this study
2	Lb-murR1	5′-TTAATTATCGTACTTATTCAGGT-3′	this study
3	Mur-SalI-F1	5′-ACGCGCATGCATGGCTGGACACAGAAA-3′	this study
4	Mur-sphI-R1	5′-ACGCGCATGCTTAATTATCGTACTTATTCAGGT-3′	this study
5	Mur-sphI-F1	5′-ACGCGCATGCATGGCTGGACACAGAAA-3′	this study
6	Mur-EcorI-R2	5′-ACATGAATTCTTAATTATCGTACTTATTCAGGT-3′	this study
7	EryB-HincII	5′-CGGTCAACATGACCACCGACGCCGCGACG-3′	this study
8	EryB-BstEII	5′-CGGGTAACCTCACTGCAACCAGGCTTCCGG-3′	this study

### Construction of recombinant plasmids

**Figure 2:**
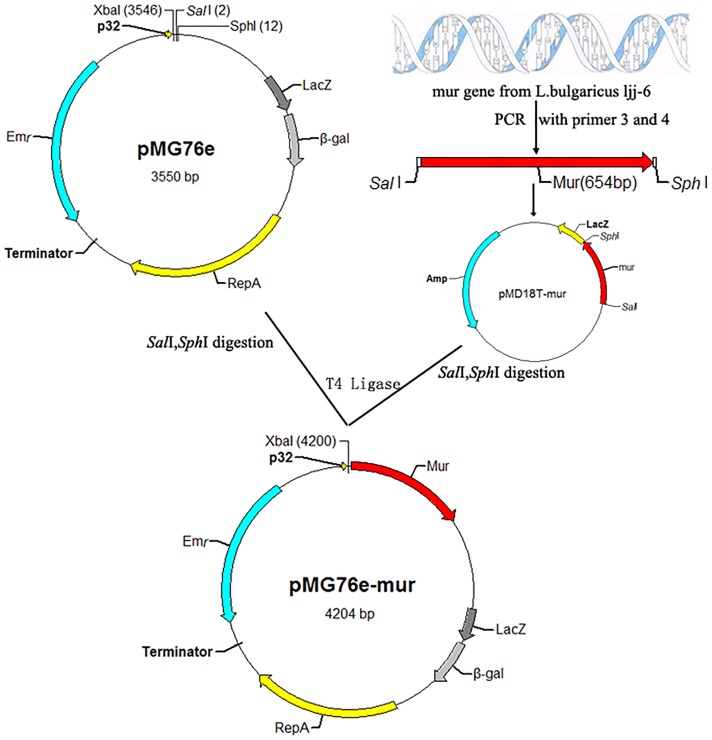
Construction of recombinant plasmids pMG76e-mur.

**Figure 3:**
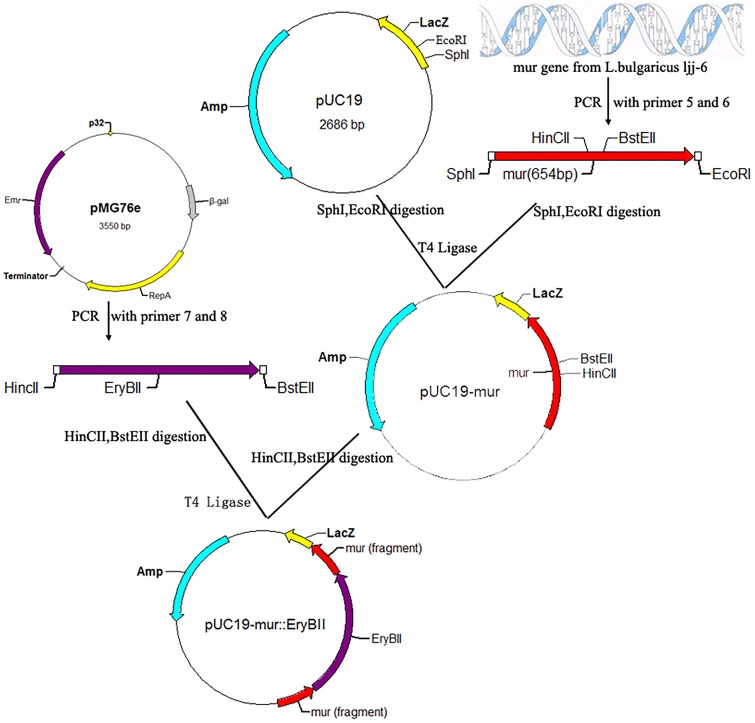
Construction of recombinant plasmids pUC19-mur::EryBII.

The *mur* gene was subcloned into pMD18-T in *E. coli* DH5α to generate the recombinant plasmids pMD18T-*mur*. Plasmid pMD18T-*mur* and pMG76e were extracted from *E. coli* DH5α, respectively. The recombinant plasmid pMD18T-*mur* and the vector pMG76e were digested with *sal*I and *sph*I, and purified with the QIAprep Spin Miniprep kit. Ligase was used to construct the recombinant of pMG76e-*mur* (Figure 2). The recombinant shuttle vector was transformed into *E. coli* DH5α by the heat-shock method and purified with the same kits. Erythromycin was maintained in the growth media at 200 ug/mL to select for positive transformants. Construction of recombinant plasmids pUC19-mur::EryBII was similar to the above method (Figure 3) [9].

### Transformation of *L. bulgaricus*

*L. bulgaricus* was transformed with pMG76e-*mur* and pUC19-mur::EryBII by electroporation according to the protocol described previously by Holo and Nes [10] and Kim et al. [11] with minor modifications. Briefly, *L. bulgaricus* was cultured in MRS medium until reaching an OD600 of 0.6–0.1. The cell pellet was then washed in deionized water and resuspended in 1/200 volume of 0.5 M sucrose containing 10% glycerol. Competent cells were added to the ligation mixture, and the mixture was treated using the Gene Pulser Apparatus (BioRad, Richmond, CA, USA) according to the manufacturer’s instructions. The electroporated mixture was immediately diluted with 1 mL of MRS broth and incubated for 2 h at 36°C and then plated on solid MRS media containing erythromycin (200 ug/mL) and incubated for 48 h at 36°C.

### Autolysis detection of lactic acid bacteria

Detection of cell autolysis rate was done using the PI-FCM method: the cultured cells in liquid were collected after centrifugation (5000×g, 5 min), followed by washing two times with PBS buffer and then resuspended. The bacterial suspension absorption value (OD_650_) was adjusted to about 1 to control the number of cells in the bacterial suspension to about 1×10^7^ cells/mL. Then 1 mL bacteria liquid was centrifuged at 4°C (3000×g, 5 min) after which the supernatant was discarded. The cells were resuspended in 1 mL PI-PBS solution (20 mmol/L). Cell suspensions were dyed by PI-PBS in a dark environment for 30 min at 4°C. A 630 nm long pass filter was used to collect the red fluorescence (FL3488 nm) and 1×10^5^ cells were collected from each sample. Test time ranged from 40 to 100 s, depending on the concentrations of the cell suspensions. Data were analyzed by CellQuest online analysis system. FCM enumerations were accurate to 10^4^ cells/mL. The rate of autolysis was defined as the ratio of the positive cell number with PI dying to the total cell number. Comparisons between groups were tested by One - Way ANOVA analysis and LSD test.

## Results

### Amplification of the full-length sequence of *mur* gene

**Figure 4:**
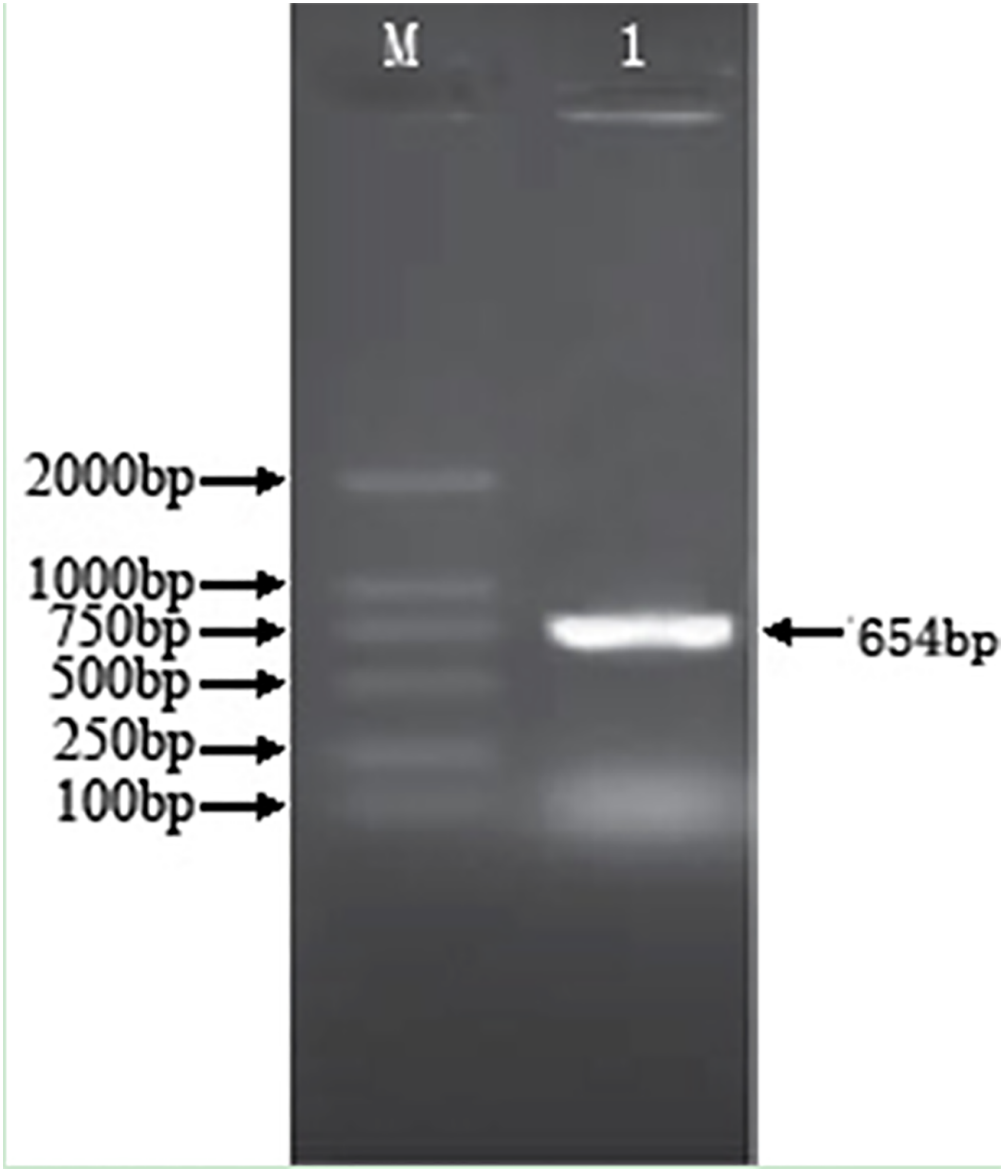
Amplification of the full-length sequence of *mur* gene. 1: The cloned 654 bp fragment from ljj-6 genome with primer 1,2; M: DNA marker DL2000.

**Figure 5:**
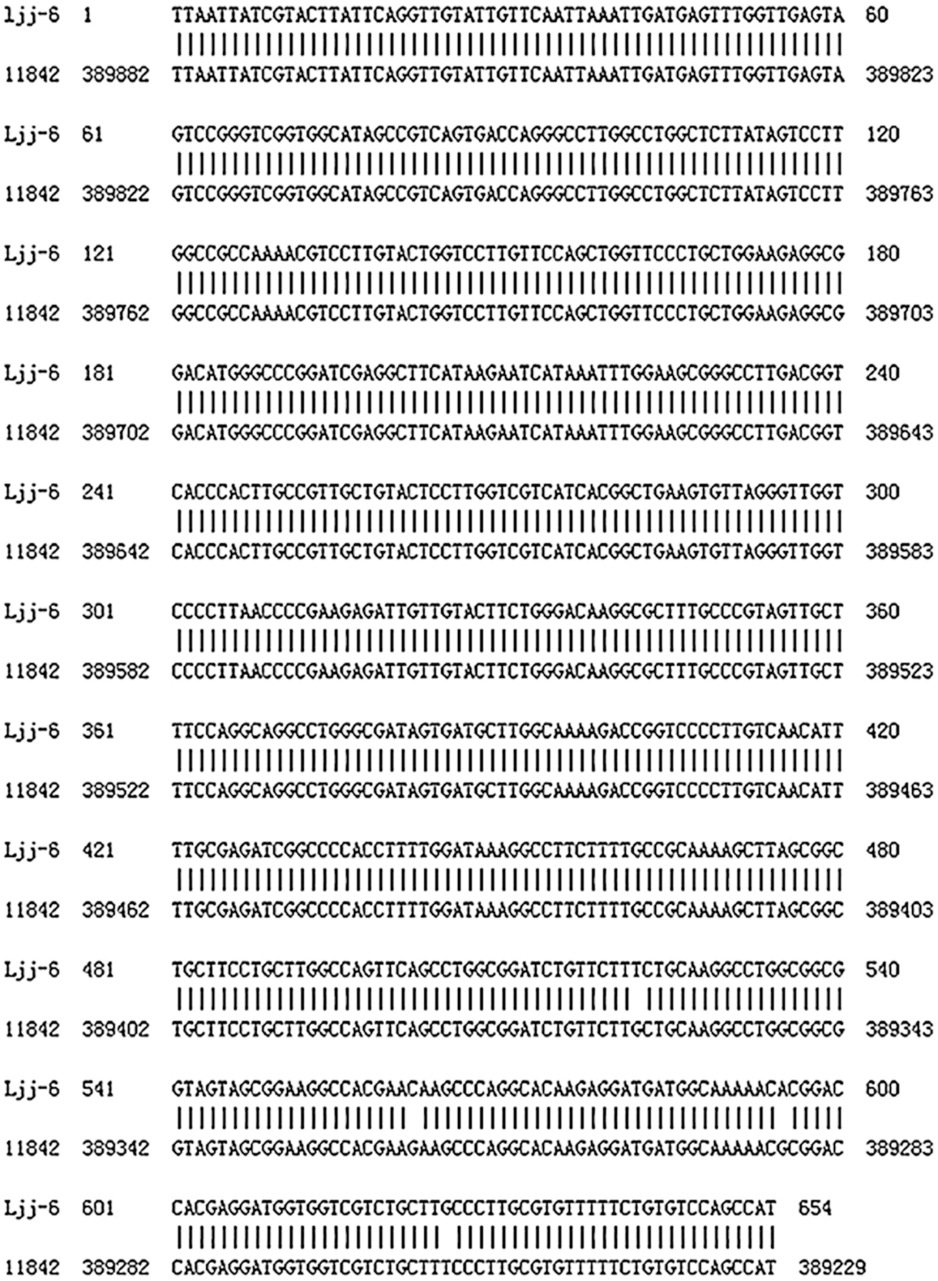
*Mur* gene fragment cloned from *L. bulgaricus* ljj-6 BLAST with *L. bulgaricus* ATCC 11842 complete genome. (Identities: 650/654).

The PCR amplification was performed with the genome DNA plate from *L. bulgaricus ljj-6*, and the amplified product was detected by 1% agarose gel electrophoresis. The results showed that a 650 bp gene fragment was obtained (Figure 4), which was consistent with other lactic acid bacteria. The sequencing results revealed four mutations in *mur* gene between ljj-6 and ATCC11842 (Figure 5). However, the amino acid sequences of these two kinds of strain were consistent, which indicated that the *mur* gene of *ljj-6* was cloned. The sequencing results were submitted to GenBank (accession number KF157911).

### Construction of recombinant plasmids

**Figure 6:**
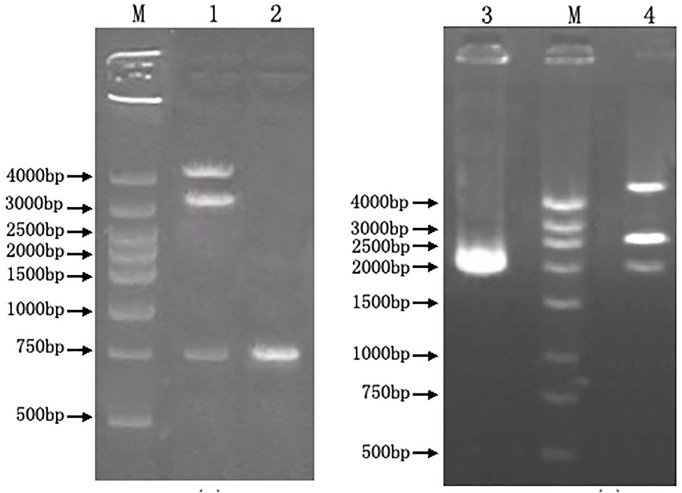
Identification of recombinant plasmids by digestion and PCR. A: pMG76e-mur; B:pUC19-mur::EryBII. 1: pMG76e-mur was digested by *Sal*I and *sph*I to 4.2 kb, 3.5 kb, 0.65 kb fragments; 2: Identification of *mur* gene from pMG76e-mur by PCR with primer 1,2; M: DNA marker DGL4000; 3: Identification of *mur* gene from pUC19-mur::EryBII by PCR with primer 1,2; 4: pUC19-mur::EryBII was digested by *Ecor*I and *sph*I to 4.7 kb, 2.7 kb, 2.0 kb fragments.

Sequence of *mur* gene approximately 650 bp in size was amplified. *Sal*I and *Sph*I restriction sites were introduced to the *mur* gene to facilitate its insertion into the multiple cloning site of pMG76e. The recombinant plasmid was amplified and isolated from *E. coli* DH5α transformants and digested with *Sal*I and *Sph*I. Analysis of the digestion mixtures by 1.0% agarose gel electrophoresis revealed that the cells contained both the 4.2 kb pMG76e-mur fragment and the 654 kb *mur* fragment, indicating that the *mur* gene had been successfully incorporated into pMG76e, in addition PCR reaction (Primer 1, 2 in Table 2) also verified this conclusion (Figure 6A).

The identification of recombinant plasmid pUC19-mur::EryB was performed using the same method as in pMG76e-mur. Analysis of the digestion revealed that the cells contained both the 4.7 kb pUC19-mur::EryBII and the 2.0 kb *mur::*EryBII fragment, indicating that the *mur::*EryBII gene had been successfully incorporated into pUC19. Additionally, PCR reaction with primers 1 and 2 also verified this conclusion (Figure 6B).

### Transformation of *L. bulgaricus*

The recombinant plasmids pMG76e-mur and pUC19-mur::EryBII were transformed into *L. bulgaricus* ljj-6 by electroporation. The electroporated cells were incubated for 2 h at 37°C and then plated on solid MRS containing 0.5 M sucrose and erythromycin (200 µg/mL). Colonies of positive transformants were visible after 48 h of incubation at 37°C. Three engineered strains (*L. bulgaricusO1-O3*) with pMG76e-mur vector and fifteen candidate engineered strains (*L. bulgaricus k1-k15*) with pUC19-mur::EryBII were screened. The fifteen candidate colonies (k1-k15) were then transferred onto MRS agar plate containing ampicillin (50 µg/ml). Possible double-crossover mutants had lost their capability to grow in the presence of ampicillin but were still resistant to erythromycin. Finally six double-crossover mutants (k7, k8, k10, k11, k12, k13) were chosen by the second round screening.

### Identification of mur gene knockout *L. bulgaricus*

**Figure 7:**
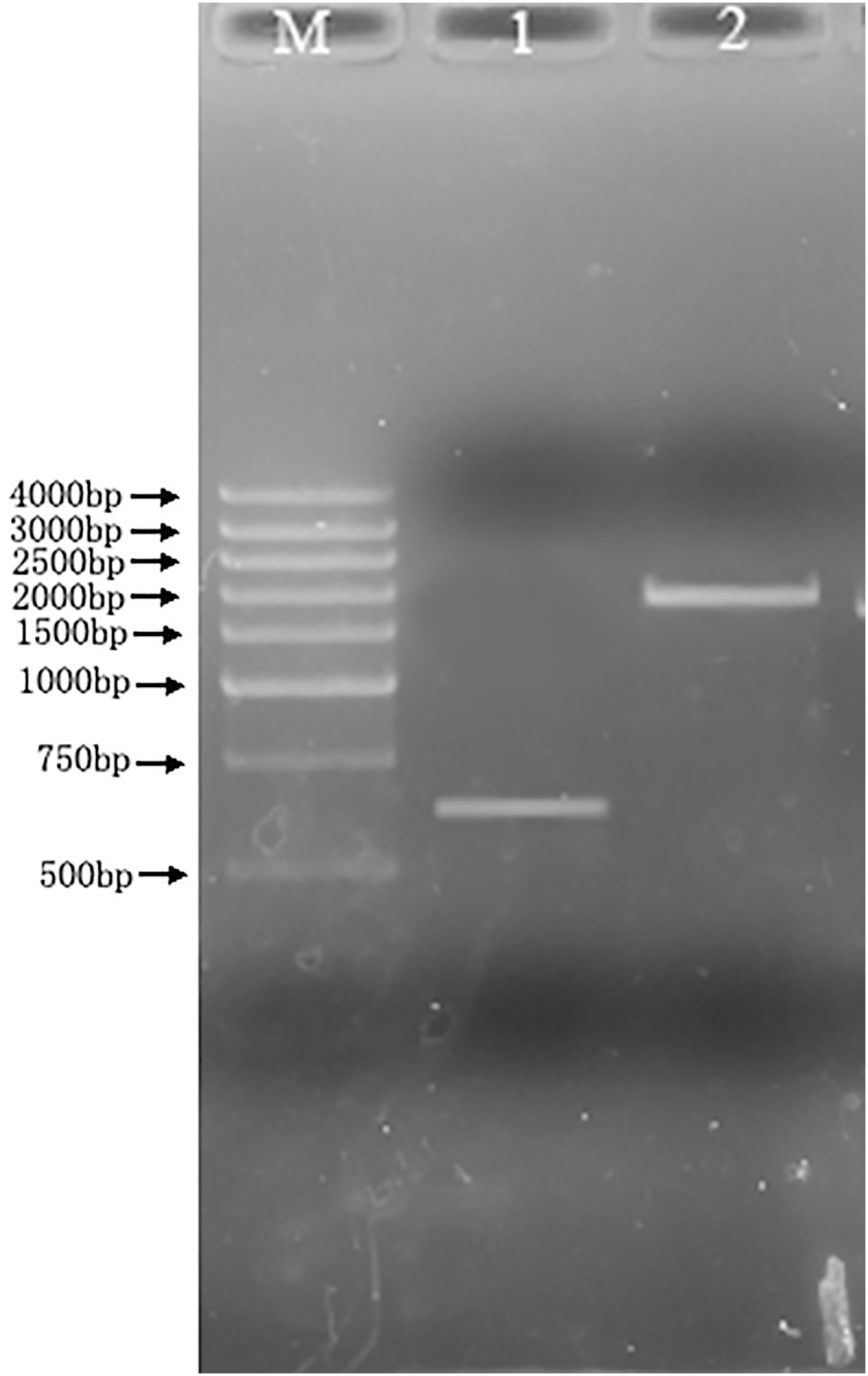
Identification of mur gene knockout *Lactobacillus delbrueckii* subsp. *bulgaricus*. M: DNA marker DGL4000; Lane 1, *Lactobacillus delbrueckii* subsp. *bulgaricus*; Lane 2, mur gene knockout *Lactobacillus delbrueckii* subsp. *bulgaricus.*

The genome DNA was extracted from the the mur knockout strain k12 which has been cultured in MRS broth for 24 hours. The PCR amplification for the mutant genome DNA and wildtype genome DNA was performed using the Primer 3 and 4 (Figure 7). The results indicated that 650 bp and 2000 bp amplified products were obtained from wildtype and mutant genome DNA respectively. The nearly 1.4 kb difference existed between two products, which was consistent with the length of inserted erythromycin resistance gene fragment (1378 bp). This implied that the mur gene knockout strain was constructed successfully through insertion of erythromycin resistance gene.

**Figure 8:**
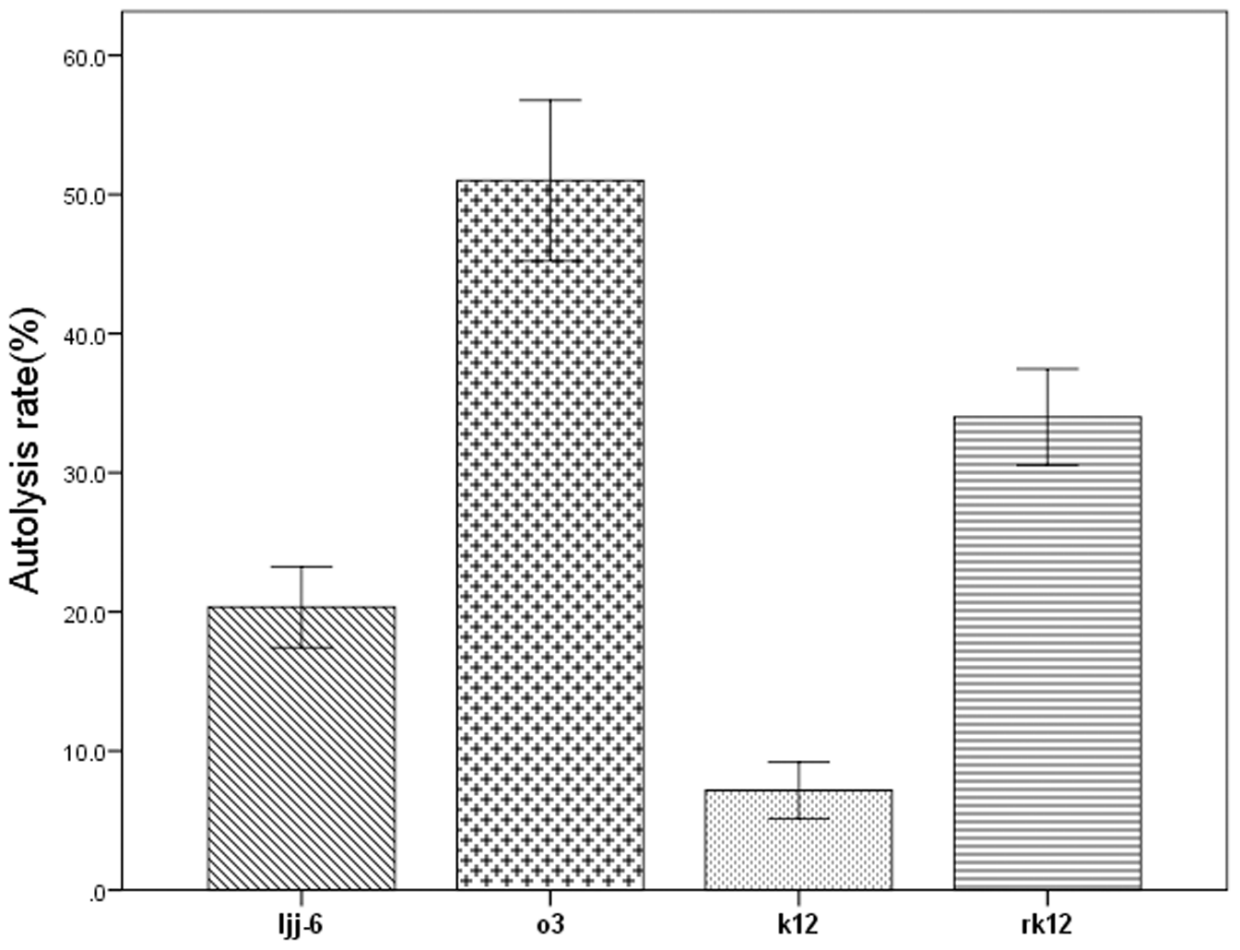
The result of autolysis detection on four strains.

The complementation experiment was carried out to exclude downstream effects of the introduced knock-out gene on other genes. To generate the *mur* reconstituted strain, pMG76e-mur was introduced into the mur deletion mutant k12 by electroporation. The mur-reconstituted strain was designated rk12. The autolysis of rk12 was detected and the result (Figure 8) showed that autolysis of strain rk12 was significantly higher compared with that of the k12 and ljj-6, this indicated that *mur* reconstituted strain restored its autolytic ability.

### Morphology observation of different autolysis lactic acid bacteria

**Figure 9:**
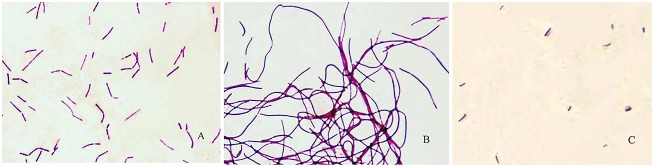
Light microscopic observation of different cellular morphology of Ljj-6 and engineering bacteria. A: *L. bulgaricus* Ljj-6 (wild type); B: *L. bulgaricus* K12(pUC19-mur::EryBII)*;* C: *L. bulgaricus* O3(pMG76e-mur). Magnification: ×1000.

Autolysis differences of lactic acid bacteria can result in significantly different variation on cell morphology. As shown in Figure 9, mycelial morphology showed a uniform rod and the boundary was clear between the cells after the wild type *L. bulgaricus* was cultured for 16 h in MRS broth at 37°C. However, for the *mur* knockout strain K12, cells were connected and became filamentous, and the boundary was blurred between the cells. For the *mur* overexpressed strain O3, the growth speed of cells was significantly slower, and the size and density of cells were smaller than wild type cells.

### Autolysis detection

The result of autolysis detection on ljj-6, o3, k12 and rk12 by flow cytometry indicated that the autolysis of the *mur* knockout strain k12 was significantly lower and autolysis of the *mur* overexpressed strain o3 was significantly higher compared with that of the wile type strain ljj-6 (Figure 8). This result suggested that the *mur* gene played an important role in autolysis of *L. bulgaricus*. On the other hand, autolytic activity in a low degree was still observed in the *mur* knockout strain, which implied that other enzymes but autolysin encoded by *mur* were also involved in autolysis of *L. bulgaricus*.

## Discussion

The rate of starter autolysis is an important factor controlling cheese ripening and flavor development. This is because many starter enzymes that affect cheese ripening, such as peptidases, lipases and enzymes that catalyze amino acid conversions, are located intracellularly (not all of the latter remain functional after being released into cheese, as they may require intracellular co-factors). After the initial breakdown of milk caseins by the lactococcal cell envelope proteinase (lactocepin; EC 3.4.21.96), autolysis of starters causing release of these enzymes usually has beneficial consequences [12], [13]. For example, it has been reported that Cheddar cheese was bitter in flavour when made using the non-autolytic starter strain L. lactis subsp. cremoris HP which contains a type-I lactocepin [14], [15]. However, Cheddar cheese made using the autolytic strain L. lactis subsp. cremoris LW1484, which contains the same specificity-type lactocepin as HP [16], [17], was not bitter [18]. On the other hand, semi–hard Saint Paulin cheese made with the autolytic starter L. lactis subsp. cremoris RD251 remained bitter due to a low level of total intracellular peptidase activity [19]. Although starter autolysis is usually beneficial, undesirable consequences such as insufficient acid production and removal of residual lactose can result if autolysis is too rapid. Autolysis of lactococci used as starter cultures in the manufacture of fermented milk results in the leakage of lipases, proteases and peptidases and other intracellular components, which play an important role in flavor development during ripening. Hence, autolysis properties of lactic acid bacteria are crucial for their applications as dairy starters.

Autolysis of lactic acid bacteria in media and buffer systems has previously been studied by Sugahara et al. [4], Østlie et al. [20], Zhang et al. [21] and Østlie et al. [22]. These studies showed that autolysis varied both among species and between strains and took place at pH 5.2 to 7.0, 30 to 40°C as optimal autolysis temperature but also moderate autolysis at 20°C and ionic strength of 0.3, all conditions comparable with the conditions in Swiss-type cheese. The pH and specificity studies Østlie et al. [20] suggest that at least two different autolytic enzymes are involved in autolysis of propionibacteria. Later, Douillard et al. [23] investigated autolysis of Lactobacillus helveticus and Propionibacterium freudenreichii in Swiss cheeses by using species-specific lysis markers and demonstrated that autolysis of this P. freudenreichii strain occurred late during ripening and was tardy. However, the importance of autolysis of propioni bacteria in cheese ripening needs further investigation since only one strain was studied for a relatively short ripening period [24].

Differences in *mur* sequences is difficult to explain the huge variation in the extent of autolysis found in different *L. bulgaricus* strains. In addition, the completed Lactobacillus bulgaricus ATCC11842 genome sequence shows the presence of several open reading frames that putatively encode cell wall hydrolases having up to 47% predicted amino acid identity to *mur* gene. These enzymes may have roles in the autolysis of *L. bulgaricus*. Further studies are needed to clarify the number and types of autolytic enzymes within different strains and species of *L. bulgaricus* and also how they work.
